# Dynamic response of the Greenland ice sheet to recent cooling

**DOI:** 10.1038/s41598-020-58355-2

**Published:** 2020-02-03

**Authors:** Joshua J. Williams, Noel Gourmelen, Peter Nienow

**Affiliations:** School of Geosciences, University of Edinburgh, Edinburgh, EH8 9XP UK

**Keywords:** Cryospheric science, Hydrology

## Abstract

The subglacial hydrological system critically controls ice motion at the margins of the Greenland Ice Sheet. However, over multi-annual timescales, the net impact of hydro-dynamic coupling on ice motion remains poorly understood. Here, we present annual ice velocities from 1992–2019 across a ~10,600 km^2^ land-terminating area of southwest Greenland. From the early-2000s through to ~2012, we observe a slowdown in ice motion in response to increased surface melt, consistent with previous research. From 2013 to 2019 however, we observe an acceleration in ice motion coincident with atmospheric cooling and a ~15% reduction in mean surface melt production relative to 2003–2012. We find that ice velocity speed-up is greater in marginal areas, and is strongly correlated with ice thickness. We hypothesise that under thinner ice, increases in basal water pressure offset a larger proportion of the ice overburden pressure, leading to reduced effective pressure and thus greater acceleration when compared to thicker ice further inland. Our findings indicate that hydro-dynamic coupling provides the major control on changes in ice motion across the ablation zone of land terminating margins of the Greenland Ice Sheet over multi-annual timescales.

## Introduction

The Greenland Ice Sheet (GrIS) has lost mass at an accelerating rate over the past two decades, with persistent mass loss observed since 1998^1–5^. Approximately 52% of this mass loss can be attributed to surface melt^6^, which increased in the late 2000s and early 2010s to levels unprecedented since at least 1900^7^. Increases in surface melt have been driven by increasing air temperatures over Greenland since the mid-1980s^7,8^ and variability in cloud cover, both of which are forced by larger scale circulation patterns^9–13^. Increased cloud-cover warms the ice sheet interior through the trapping of longwave radiation^14,15^, whereas a reduction in summer cloud cover since 1995 has driven enhanced melt in the ablation zone through increasing the shortwave flux^10,16^. Moreover, the seasonal migration of the snowline causes the exposure of dark bare-ice, decreasing the albedo of the ice surface and reducing meltwater re-freezing, further driving surface melt and runoff^17^. Alongside changes in surface mass balance, roughly 48% of mass loss is due to increases in ice discharge through Greenland’s marine terminating outlet glaciers^6^. However, the dynamic response of the ice sheet to variability in surface mass balance and ocean conditions remains a large source of uncertainty in projecting future sea level rise^18^.

Land-terminating margins are isolated from processes acting at the ice/ocean boundary, and thus provide ideal study sites for investigating how the ice-sheet responds to atmospheric, and thus surface melt forcing^19,20^. This is particularly prescient as the largely land-terminating margin in South West Greenland exhibits a strong and sustained negative mass balance^21,22^, and is projected to make a greater contribution to sea level rise with continued atmospheric warming and associated increases in surface runoff^23^.

Each summer, surface meltwaters drain from the ice-sheet surface to the bed via moulins and crevasses^24–27^ where their impact on friction at the ice-bed interface is fundamentally important in controlling ice velocity^28,29^. Whilst initial research postulated that as these seasonal meltwaters drain to the base of the ice sheet, they would pressurise the basal hydrological system, reduce friction at the bed and so enhance glacier sliding^30^, other studies have argued that variability in meltwater input, rather than the volume itself, is more critical for driving ice acceleration^31,32^.

Both short-term and sustained increases in surface meltwater delivery to the glacier bed drive an increase in basal water pressure above the ice overburden pressure, reducing friction at the bed and so forcing a transient acceleration^32–34^. These inputs force a morphological switch from an inefficient, distributed subglacial drainage system to an efficient, channelised system when and where subglacial discharge is sufficiently turbulent to open subglacial channels^26,32,35,36^. This introduces a negative feedback whereby as the drainage system capacity increases in response to enhanced meltwater input, basal water pressures decrease as subglacial channels allow the efficient evacuation of subglacial water^26,37^, forcing a deceleration of the ice later in the melt season^27,32,38^.

More recently, research has focused on whether this hydro-dynamical coupling of ice flow at land-terminating margins results in a long-term trend in ice-motion in response to long-term increases in surface melt^39,40^. Multi-annual ice velocity slowdowns in southwest Greenland since the early-mid 2000s have been reported by numerous studies based on both GPS data^41–43^ and large-scale satellite-derived observations^40,44^. Ice velocities from GPS stations along a transect in Southwest Greenland, extending ~150 km inland from the margin between surface elevations of 340 m and 1850 m above sea level (a.s.l), show a 10% average slowdown from 1991–2007^41,42^, coincident with increasing surface melt, and GPS data at North Lake show a slowdown of −0.9 ± 1.1 m yr^−2^ from 2006–2014^43^. Over a much larger 8000 km^2^ region of Southwest Greenland, Tedstone *et al*.^40^ showed that ice velocity had decreased by 12% in 2007–14 compared to 1985–94, despite a 50% increase in surface meltwater production, with ice velocity decreasing by 1.5 m yr^2^ between 2002 and 2014. This long-term slowdown is attributed to the expansion of subglacial channels, both up-glacier and in their dimensions, as a result of the long-term increase in surface melt, enhancing the drainage of waters from the more extensive distributed component of the subglacial drainage system, thus reducing regional basal water pressures and so ice velocities^38,40^. The distributed component of the subglacial drainage system encompasses varying degrees of connectivity, and it is hypothesised that the reduction of basal water pressures in the weakly-connected areas of the drainage system specifically^27,39^ are critical to the observed slow-down as their recharge is slow (on the order of years^39^), resulting in widespread and extended depressurisation and so increased basal traction.

Since the record surface melt in 2012^3^, a period of relative stability in mass balance^45^ has been observed across Greenland, with 2017 having the lowest maximum surface melt extent since 1996^46^. This stability is coincident with positive Arctic and North Atlantic Oscillations, promoting cyclonic conditions thereby reducing incoming solar radiation and enhancing precipitation^46^. Given that the proposed hydro-dynamic mechanism for a long-term velocity slowdown requires a continual increase in surface melt^39,40^, it would be expected that ice motion would respond to a sustained change in surface run-off and begin to stabilise or accelerate as surface melt decreases and the distributed subglacial hydrological system re-pressurises. Under reduced surface melt forcing, we would expect the up-glacier extent of efficient subglacial channels to decrease, allowing regions of the distributed drainage system that were, in previous years, drained by efficient channels to re-pressurise through the gradual recharge of meltwater via basal melting. Numerous GPS data show that this process occurs on a seasonal timescale, whereby following the deceleration of ice motion to a minimum in the late melt season, measured ice velocities show a gradual increase over the following winter^41,47,48^; this process has not however been observed to-date on a multi-annual timescale. This study therefore extends the West Greenland ice velocity time series, both spatially and temporally, in order to investigate how ice motion has responded to recent reductions in surface melt forcing, with the ultimate aim of improving our understanding of the mechanisms driving ice sheet motion.

Here we present observations of ice velocity from 1992–2019 across a predominantly land-terminating area of ~10,600 km^2^ in SW Greenland, extending ~300 km along the margin and ~50 km inland to an elevation of 1300 m. Our study region is considerably larger than that of Tedstone *et al*.^41^, extending a further ~120 km to the south. We apply feature tracking to 2665 pairs of Landsat scenes, separated by 352–384 days, over 14 path/row combinations. Subsequently, we derive robust ice velocity and uncertainty estimates^49,50^ for periods of 1, 2 and 4 years to construct a time series from 1992 to 2019, and assess the spatial distribution of velocity change between 1992–2003 and 2003–2012, and 2010–2012 and 2017–2019. Finally, we assess the implications of our results for future land-terminating ice-sheet motion in a warming climate in light of the dynamic response of the study region to the recent variability in meltwater forcing.

## Results

### Spatial pattern of velocity change

**Figure 1:**
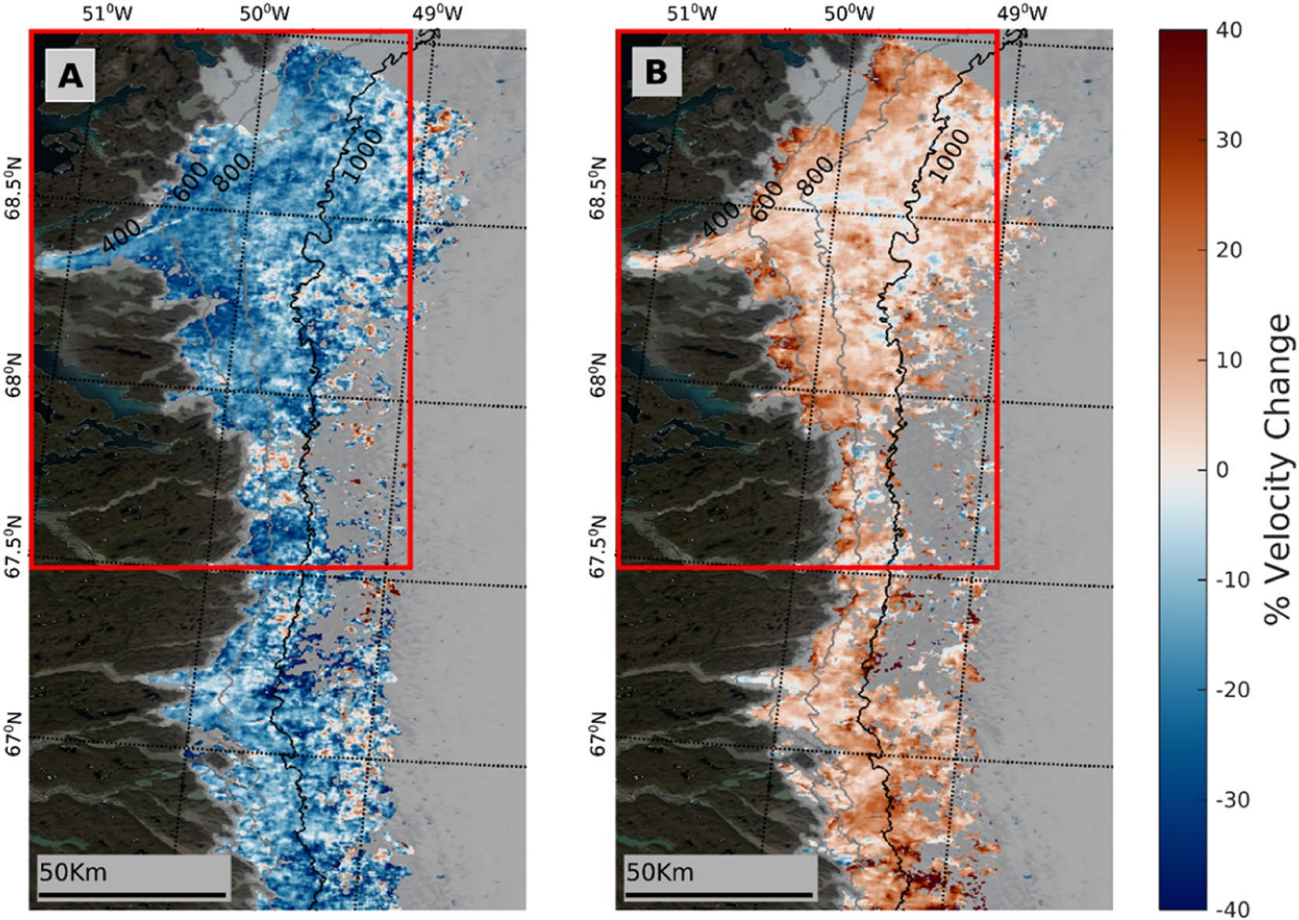
Spatial change in ice velocity (%) between the **(A)** 2003–2012 and 1992–2003 and **(B)** 2017–2019 and 2010–2012 reference periods. Data above 1300 m a.s.l. are filtered out in order to remove spurious points that characterise higher elevations. The red rectangle denotes the region studied in Tedstone et al.^40^. Ice surface elevation contours (grey lines) are from Howat et al.^88^, with the 1000 m contour bold to make clear the area across which the velocity time series was calculated (Fig. 3). The two tidewater glaciers to the north of the study region are masked out as they are undergoing different dynamic processes to the rest of the region. The base image is a MODIS (Terra) corrected reflectance image from EOSDIS NASA Worldview (https://worldview.earthdata.nasa.gov/).

**Figure 2:**
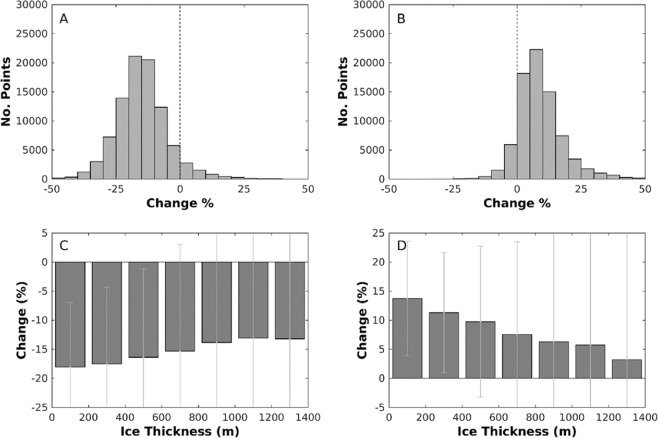
Histogram of ice velocity change across the study region and percentage change across ice thicknesses for the two changemaps displayed in Fig. 1. Plots A and C relate to Fig. 1A (2003/2012–1992/2003), and plots B and D relate to Fig. 1B (2017/2019–2010/2012). **(A)** Percentage change in ice velocities across the region displayed in Fig. 1A in 5% bins. **(B)** Percentage change in ice velocities across the region displayed in Fig. 1B in 5% bins. **(C)** Median percentage change in each 200 m ice thickness band between 0 m and 1400 m for the changemap displayed in Fig. 1A. (**D**) Median percentage change in each 200 m ice thickness band between 0 m and 1400 m for the changemap displayed in Fig. 1B. The error bars display the interquartile range. Ice surface elevation data is from Howat *et al*.^88^, and ice thickness data are from Morlighem *et al*.^89^.

Ice velocity displays a clear slowdown across the study site (Fig. 1A) between the periods 1992–2003 and 2003–2012, with 93.1% (9895 km^2^) of the region exhibiting reduced velocity in the latter period, and a mean regional slowdown of ~15.3% (Fig. 2A). The slowdown is greatest at lower ice thicknesses and decreases in magnitude inland as surface elevation and ice thickness increase (Fig. 2C), although deceleration characterises all ice thicknesses assessed and extends further inland than in previous work^40^. We would expect this to be the case as at higher elevations further inland, lower surface melt rates, thicker ice and shallower surface slopes slow channel growth, allowing subglacial water pressures to remain higher in smaller subglacial channels^26,49,50^.

Subsequently, we observe a mean region-wide acceleration of ~7.9% between the periods 2010–2012 and 2017–2019 (Fig. 1B), with 89.6% (8218 km^2^) of this second change map exhibiting increased velocity during the latter period (Fig. 2B). This acceleration in ice motion is proportionally largest at lower ice thicknesses and decreases as ice thickness increases but is observed across all ice thicknesses studied (Fig. 2D).

### Ice velocity and surface melt/runoff time series

**Figure 3:**
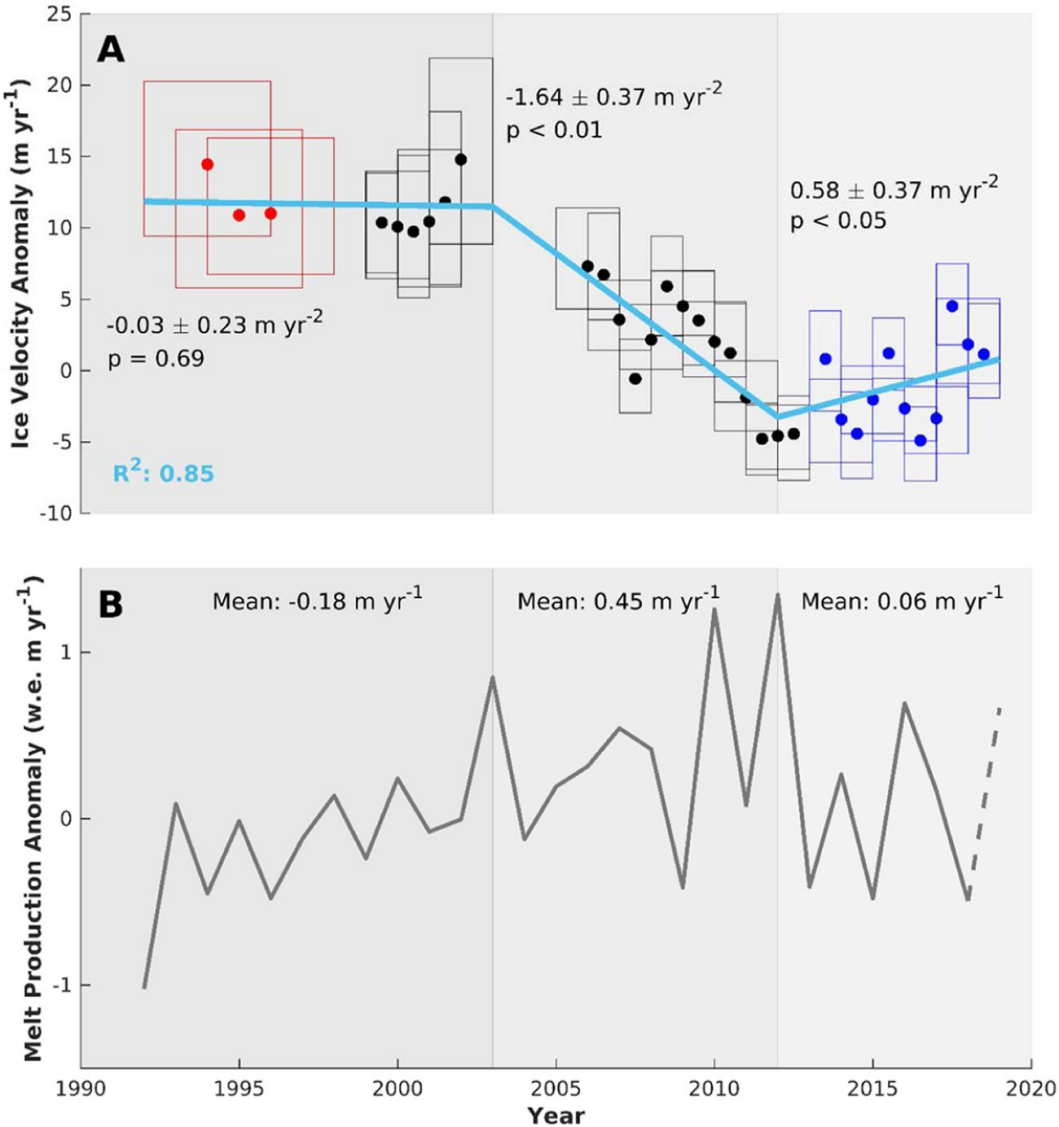
(**A**) Median ice velocity anomalies (m yr^-1^) during each period calculated by sampling the common pixels between all velocity fields in the times series. Red boxes indicate Landsat 5 data, black boxes indicate Landsat 7 data, and blue boxes indicate Landsat 8 data. The width of each box corresponds to the total time period of the pairs in Landsat scenes fused for each period. The height of each box corresponds to the interquartile range and the light blue line displays the trends in ice velocity computed by a segmented linear regression (see Methods and SI). **(B)** Annual modelled surface melt production anomaly (grey)^7^ in water equivalent (w.e.) m yr^-1^ (see Methods and SI). A dotted line is used to display the data for 2019 as this was incomplete at the time of processing. The background shades are used to differentiate the three distinct periods of dynamic behaviour.

**Figure 4:**
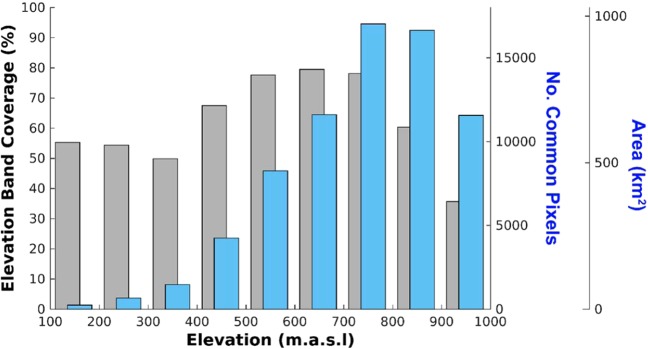
Percentage coverage of each 100 m elevation band by common sampling pixels (grey, left axis) and the altitudinal distribution in the common sampling pixels used in the computation of median ice velocities (blue, right axes).

To investigate changes in ice motion (Fig. 3A), we follow recent work by assessing anomalies in annual velocity (see SI) whereby only pixels common to all the velocity fields presented in the time series are used in the computation of the median velocity anomalies^50^. Most of the common points fall between 600 m and 1000 m elevation (a.s.l.) due to the hypsometry of our study region (Fig. 4), although we observe >50% coverage in each 100 m elevation band between 100 m and 900 m. In total, we compute velocity anomalies across 71703 common measurements (4130 km^2^), an order of magnitude greater than previous work^40^ (their Fig. 2C).

Over the time period from 1992 to 2019, the ice velocity anomaly time series reveals a mean slowdown of −0.72 ± 0.08 m yr^−2^ (R^2^ = 0.70, p < 0.01). The quality of the fit is further improved via break point analysis (see SI) which recognises three statistically distinct periods based on the pattern of velocity changes (R^2^ = 0.85 (Fig. 3A)). Between 1992 and 2003, we observe a period exhibiting no significant trend (−0.03 ± 0.23 m yr^−2^, p = 0.48). A substantial slowdown of −1.64 ± 0.37 m yr^−2^ (p < 0.05), starting around 2003 (but which could range from 2002–2004 due to a gap in our data), occurs until 2012, after which the most recent period of ice motion exhibits a significant increasing trend (0.58 ± 0.37 m yr^−2^, p < 0.05).

We use the regional climate model MAR v3.10^7,9^ to calculate the mean surface melt production anomaly during the three distinct periods identified in our ice velocity anomaly time series; 1992–2003, 2003–2012, and 2012–2019 (Fig. 3B). Mean surface melt production anomalies across our study region rose by ~30% from −0.18 m yr^−1^ to 0.45 m yr^−1^ (w.e.) (~2.15 m yr^−1^ to ~2.77 m yr^−1^ (w.e.)) between 1992–2003 and 2003–2012. Following this period of sustained higher surface melt production, a ~15% decrease to 0.06 m yr^−1^ (~2.39 m yr^−1^) is observed for 2012–2019, when compared to the 2003–2012 mean. The period of ice velocity decrease therefore coincides with a period of enhanced ice surface melt, while rates of constant and accelerating ice velocity occur during periods of lower surface melt.

Previous work has argued that there is a significant relationship between antecedent surface melt production and ice velocity^40^. More recent work however suggests that this may be a statistical construct^43^, whereby as more antecedent melt years are included in the average value, R^2^ tends further towards 1 (Supplementary Fig. 12) as the data becomes increasingly smoothed and the individual points become less independent. With only one period of sustained slowdown, we are unable to test whether ice velocity slowdown is in response to a gradual increase in surface melt or to the passing of some surface melt threshold. Moreover, the response times of ice velocity to increases and decreases in surface melt forcing appear to differ – surface melt displays a long-term increasing trend from the early-mid 1990s before ice velocities begin to decrease in ~2003, whereas ice velocities stabilise and begin accelerating almost instantly in response to the large reduction in surface melt forcing from 2013 onwards. To investigate the impact of year-to-year variability in surface melt production on year-to-year velocity, we calculate a linear regression through detrended velocity and melt production anomaly time series (Fig. S13), which gives an R^2^ of 0.08 (p = 0.11), indicating that there is no significant relationship between annual ice velocity and annual runoff, consistent with earlier work^41,43,48^.

## Discussion

Changes in ice thickness and surface gradient can contribute to changes in ice motion through their associated impacts on driving stress. Thinning at the margins of the Greenland Ice Sheet has been observed since the early 1990s^2^, and from 1992–1998, the South West Greenland land-terminating sector thinned by ~0.02–0.4 m yr^−1^ ^51,52^. A period of stability characterised the late 1990s and early to mid-2000s^52^, followed by rapid thinning (~ 1–1.5 m yr^−1^). Whilst some datasets assume a constant thinning of a similar magnitude through to 2014^53^, recent work points to a maximum thinning rate in 2012, after which thinning has continued at a more moderate rate^54^, albeit with differences in thinning trends between drainage basins^52,55^. Prior modelling work in our study region^40^ indicates that changes in driving stress due to thinning could only explain 17–33% of the slowdown signal beyond 10 km from the ice-margin, and none of the signal beyond 50 km. This modelling work applied 20 m of thinning over 30 years, equating to a thinning rate of −0.66 m yr^−1^. Outside of the period 2007–2011, there is no evidence for thinning rates greater than this during our study period^51–53,56^, and we thus do not believe that thinning rates could be responsible for more slowdown than already reported in Tedstone *et al*.^40^. Moreover, continued thinning from 2012 would be expected to continue to reduce ice motion, which contrasts with the acceleration in ice velocity observed during this period. As such, these findings imply that most of the ice velocity signal is controlled by processes operating at the ice-bed interface.

The subglacial hydrological system exerts a critical control on ice motion at land-terminating margins of the Greenland Ice Sheet^38,57^. Our results provide support for a previously postulated mechanism^40^, whereby under a sustained multi-annual increase in surface meltwater production, the subglacial drainage system is characterised by both a gradual increase in the extent of channelisation, as well as the time during which these channels remain open and are thus able to evacuate water from the surrounding distributed drainage system^58^. These conditions promote the drainage of waters from weakly-connected^39^ regions of the distributed drainage system, thereby reducing basal water pressure and associated basal lubrication and hence ice velocity over longer (decadal) timescales as effective pressures increase. Whilst it is hypothesised that increasing runoff to the bed will increase sediment pore water pressure, resulting in reduced sediment shear strength, increased sediment deformation and thus enhanced ice flow^59^, such behaviour contrasts with the observations reported here and elsewhere^40–42,44^. Consequently, our results suggest either that extensive layers of subglacial till are not ubiquitous across our study region or if they are present, the tills have not deformed pervasively in response to the sustained period of enhanced meltwater input.

Surface melt production in our study region peaks in 2012, decreasing thereafter (although surface mass balance remains negative), consistent with trends observed across the ice-sheet^46^. Concurrent with decreasing meltwater runoff, we observe an acceleration in ice velocity. During the period of sustained high melt, the drainage of basal waters from a larger area of the distributed drainage system causes a gradual depressurisation of the background water pressure with an associated increase in effective pressure. Reduced surface meltwater production would result in seasonally less extensive and smaller subglacial channels and consequently these channels will undergo faster creep closure^32^. This results in a reduced time-period over which the main subglacial channels can evacuate water from the surrounding weakly connected, hydraulically distributed regions. As water pressure is no longer being systematically reduced, the background water pressure can increase through a gradual re-pressurisation of the subglacial hydrological system, as meltwater can recharge from basal melting without being evacuated by efficient subglacial channels.

Effective pressure (*N*) is calculated as *N = Pi − Pw*, where *Pi* refers to the ice overburden pressure and *Pw* to the basal water pressure^32,60^. Repressurising the subglacial hydrological system causes basal water pressure (*Pw*) to increase, thus reducing effective pressure and causing ice to accelerate. As effective pressure is a function of ice thickness, for the same increase in basal water pressure we would anticipate acceleration to be greater where ice is thinner. Although we expect channel closure to occur earlier and faster under thicker ice (due to higher ice overburden pressure)^26,49,50^, modelling studies suggest that once channel pressure falls to 90% of overburden pressure, there is less than 2 days difference in closure time for channels with a cross-sectional area of 10 m^2^ compared to <1 m^2^ ^50^. Moreover, it has been suggested that maximum channel closure rates are similar at both marginal and interior sites^49^. Consequently, we argue that any variation in repressurisation due to channel closure rates will only have a small impact upon effective pressures when compared to variability in ice thickness (which ranges from 0–1400 m across our study area). As a result, we hypothesise that the observed acceleration is greater for marginal regions of thinner ice due to the impact of basal meltwater recharge on water pressure in these areas offsetting a larger proportion of the ice overburden pressure (Fig. 2D).

The inland limit and spatial extent of efficient channel formation is subject to considerable debate^38,61^. Borehole and tracer studies and ice velocity records have been used to infer channels extending 40–80 km inland^19,25,26,34,62–64^, with high flow velocities of traced waters and the rapid transmission of pulses of meltwater from the ice sheet surface to the margin indicating efficient drainage^38,65^. These observations are supported by modelling studies, whereby efficient channels have been modelled up to 50 km inland, under 900 m thick ice^66,67^. The spatial extent of channelisation is influenced by the distribution of surface-to-bed connections, with high moulin density conducive to widespread and rapid channel development^68^. Further inland, ice thickness increases, surface slopes become shallower and runoff production decreases, leading to enhanced creep closure due to higher effective pressures and reductions in hydropotential gradients and wall-melting^32,61,69,70^. Under these conditions, modelling studies suggest that at a certain elevation and ice-thickness, efficient subglacial channels will not develop^49,50^ in which case melt and ice-velocity are expected to scale positively^71^. Velocity measurements 140 km from the margin^71^, within the accumulation area of the Leverett catchment, indicate a 2.2% increase in annual velocity between 2009 and 2012. In our study, we are unable to determine whether a positive change in ice velocity has occurred at elevations above 1300 m (a.s.l.) due to increasing noise in our dataset further inland. However, GPS observations reveal reduced velocities to at least 80 km inland (at ~1500 m a.s.l) in the years of record surface melt in 2010^48^ and 2012^37^ when compared to 2009.

Whilst multi-annual ice velocity slowdowns in southwest Greenland since the early-mid 2000s are observed here and in a number of other studies^40–44^, differences between studies exist regarding the magnitude of slowdown, and the proposed mechanism(s) driving this dynamic change. For example, whilst GPS data show a slowdown of −0.9 ± 1.1 m yr^−2^ at North Lake from 2006–2014^43^, satellite radar data from 2000/01 to 2016/17 reveal no significant trend (−0.2 m yr^−2^, p = 0.62)^44^. At a regional scale, this radar data suggests a weaker slowdown trend than that reported either here, or by Tedstone *et al*.^40^ (−1.5 m yr^−2^ from 2002–2014, −12% between 1985–1994 and 2007–2014), across the same study area, with little in the way of significant changes between 2001/01 and 2016/17 across much of the southwest margin^44^. Methodological differences between the studies may affect the conclusions drawn as this study and that of Tedstone *et al*.^40^ applied feature tracking to image pairs either side of an annual baseline (352–400 days in Tedstone *et al*.^40^, 352–384 days here), whereas Joughin *et al*.^44^ composed mosaics of winter velocities, comprised of data sampled between September and May. Regardless, this raises the possibility that the previously observed slowdown^40^ may be related to some local process, rather than regional-scale surface melt forcing^44^, for example water piracy as a result of dynamic thinning on Jakobshavn Isbrae has been suggested as a driver of slowdown in the north of the West Greenland land-terminating sector^44^. However, by extending the study area ~120 km to the south, and by increasing the number of common pixels used in our time series by an order of magnitude compared to that of Tedstone *et al*.^40^, we extend confidence in the observed slowdown of ice motion from the early-mid 2000s. Moreover, by calculating an ice velocity anomaly^72^, we remove the influence of any biases between sensors from the observed trends, further increasing confidence that this sector of the ice sheet underwent significant deceleration during this period.

A variety of models exist to simulate the subglacial drainage system and its impact on ice dynamics in response to increasing meltwater fluxes in a warming climate^73^. However, whilst current models can reproduce observed dynamics of Greenland’s land-terminating margins over days/weeks^32,50^, on a seasonal timescale^39,67,74^, and across several years^66,75^, there is a need to consider the longer, decadal and multi-decadal response of ice motion to surface meltwater forcing in order to better reproduce observed dynamics and project future change. On a decadal scale, recent modelling predicts increased ice-motion and thus ice flux into the ablation zone under enhanced summer melt within 44 km of the ice-margin^76^ (their Figure 5C). These predictions contrast with the results presented both here and from other GPS and satellite-derived observations^37,40–44^. Further work is therefore required to investigate what aspects of the model set-up or framework cause the modelled ice-motion to accelerate on a multi-annual timescale in response to increasing surface runoff, in contrast to numerous observations reported in this and other studies. Whilst we do observe reduced slowdown as ice thickness increases, our results and those of others show that ice motion within the ablation area has slowed as a result of increased surface runoff, and we do not observe any appreciable or spatially extensive increases in ice velocity as a result of an extended period of high surface melt. We therefore argue that in order to better represent ice dynamical processes and project future changes in coupled hydro-dynamic models, the models must better utilise the available data from field and satellite observations both in model set-up and for assessing model performance, and there is a need for changes in the numeric representation of processes, including ice-hydrology coupling.

Our findings demonstrate that ice at surface elevations below 1300 m (a.s.l.) in the South West land-terminating region of the GrIS underwent deceleration during the mid to late-2000s and early-2010s with subsequent acceleration following sustained reductions in surface melt production, with this acceleration proportionally greater in areas of thinner ice. We hypothesise that this recent acceleration is driven by increases in basal water pressure offsetting a larger proportion of ice overburden pressure, and thus causing a greater reduction in effective pressure under thinner ice. This behaviour has not been observed previously on a multi-annual timescale. Whilst we have demonstrated that changes affecting the subglacial hydrological system are the most likely driver of ice velocity change in our study region, we are unable to conclusively state whether antecedent melt production is the predominant factor controlling ice motion, or whether some threshold in the amount of melt is required in order to drive a change in velocity. However, it is clear from our results that the period of ice deceleration occurred during a period of sustained high melt production, and ice motion accelerated subsequently as melt production decreased to consistently lower values from 2013 onwards. Moreover, we observed no indication of an ongoing slowdown once surface melt production exhibited a sustained decrease. Thus, the behaviour observed supports a process-based understanding of the links between hydrology and ice dynamics in this land-terminating sector of the Greenland ice sheet. The results displayed in this study show that the observed trends in ice velocity are not statistical artefacts or the result of biases between sensors^72^. Further, we see no evidence of speed-up at the elevations studied under a warming climate, irrespective of the bed conditions. Future work is required to improve observations of change at higher elevations, and at other land-terminating sectors of the ice sheet, in order to assess the extent to which these processes operate on an ice sheet scale.

Furthermore, while the results presented here relate specifically to land-terminating margins, it has been argued that the dynamics of tidewater glaciers are a product of both oceanic and atmospheric forcing^77,78^, with the latter potentially driving a positive feedback as a result of its influence upon fjord water circulation and thus submarine melt^77,79,80^. This positive feedback contrasts with our findings at land-terminating margins, indicating that at an ice-sheet scale, the relationship between hydrology and ice dynamics is complex and spatially variable. Since the extent to which surface melt processes impact on tidewater glacier dynamics remains uncertain, analysis of existing large-scale, multi-decadal datasets^5^ at marine-terminating margins should be undertaken with respect to surface melt change, in order to better project the future response of the Greenland ice sheet to changing atmospheric and oceanic conditions.

## Methods

### Remote sensing of ice velocity

We apply optical feature tracking^40,81^ to all Landsat pairs for the 14 path/row combinations that intersect our study region (66.41 to 69.52N, −51.78 to −45.45 W, Figures S1–S3). A pair length of 368 ± 16 days is used to minimise the impact of any seasonal variability in interannual ice velocity (Fig. S4). We use oriented correlation, matching the feature of gradient orientation for each pixel^82^, and use a combination of spectral bands at wavelengths ~0.52–0.69 μm (bands 2 + 3 for Landsat 5 and 7, 3 + 4 for Landsat 8). We enhance the images by applying a principal component analysis to these bands, and subsequently, a high-pass gaussian filter is used to enhance surface features such as crevasses and reduce the impact of temporally stable features relating to the basal topography^40,81^. We use a reference window of 80 pixels and set the search window based upon prior velocity estimates^83^. Following Tedstone *et al*.^40^, we apply a median coregistration to the output velocity fields in order to remove errors associated with georeferencing.

The coregistered velocity fields are then fused via a spatio-temporal median over annual or multi-year periods. Low-quality velocity estimates are removed through filtering by a threshold signal-to-noise ratio value (snr threshold = 6), identified by calculating the value beyond which the median absolute deviation of velocities over stable ground becomes asymptotic^81^. The final velocity field for each time period is composed of the median value of all velocity fields within the time period at each pixel. To calculate uncertainty at each pixel, we compute a 1σ confidence interval for each component of the velocity field in the form:

$$\sigma =\frac{k}{2}\cdot \frac{MAD}{{N}^{\alpha }}$$

Where MAD is the median absolute deviation over stable ground, N is the number of velocity fields used in the computation of the median velocity, σ is the 1σ confidence interval, and k and α are parameters determined for each time period from the stable ground velocity which is known to equal zero. This relationship is extrapolated on-ice using the appropriate values of MAD and N at each pixel. Following Tedstone *et al*.^40^, we discard pixels with σ > 60 myr^−1^ from the subsequent analyses. In addition, we mask out the tidewater glaciers to the very north of the study region, limit our analysis to pixels below 1000 m above sea-level, and retain only the velocity fields with an area coverage above 9250 km^2^. The median velocity is subsequently calculated across the 71703 pixels common to all the remaining merged velocity fields (Fig. S5) in order to avoid spatial bias influencing the change signal.

### Anomaly-based time series

Recent work indicates that velocity magnitude has a biased mean, with this bias increasing with the standard deviation of the velocity components (and so with noise), causing an artificial negative velocity trend^72^. To mitigate this effect, we follow the velocity anomaly approach of Dehecq *et al*.^72^. First, we calculate a mean of all velocity pairs covering the period 1992–2019 described as V_0_. The velocity anomaly is defined as the value of the difference vector V_t_ − V_0_ projected on the mean velocity vector:

$$dv=\,\frac{({V}_{t}-\,{V}_{0})\cdot {V}_{0}}{\Vert {V}_{0}\Vert }=\frac{({V}_{x,t}-{V}_{x,0}){V}_{x,0}+({V}_{y,t}-{V}_{y,0}){V}_{y,0}}{\Vert {V}_{0}\Vert }$$

The result of this approach is to centre the noise distribution symmetrically around zero (Fig. S6) such that there is no bias in the mean value, removing any artificial slowdown trends due to variability in noise magnitude between sensors^72^. The resultant velocity anomaly fields are displayed in Fig. S7.

To assess the long-term trends in our time series, we first compute a simple linear regression through the data, and then test whether the data can be divided into three statistically different segments (Figures S8–S10) using the non-parametric Mann-Whitney Wilcoxon Test. We select the pair of breakpoints with the lowest RMSE, and that are statistically significant with 99% certainty, which gives breaks at 2003 and 2012.

### Spatial trends in ice velocity

We construct change maps displaying the percentage ice velocity change between the following periods; 1992–2003 and 2003–2012, 2010–2012 and 2017–2019. For each pair, median ice velocity for each period was calculated through fusing all constituent velocity fields via a spatio-temporal median, and uncertainty of the percentage change was calculated through a linear combination of the uncertainties of each period:

$$\sqrt{{{c}_{1}}^{2}+{{c}_{2}}^{2}}$$

where c_1_ is the first period (i.e. 1992–2003) and c_2_ is the second period (i.e. 2003–2012). We remove pixels with uncertainty greater than 60 m yr^−1^. We also filter by Velocity Vector Coherence (VVC)^81^, which follows the form:

$$VVC(i,j)=\frac{\left\Vert {\sum }_{t\in T}\mathop{\to }\limits_{V}(i,j,t)\right\Vert }{\left\Vert {\sum }_{t\in T}\mathop{\to }\limits_{V}(i,j,t)\right\Vert }$$

where T is the set of N velocity estimates V(i,j,t) merged to obtain the median velocity $\bar{V}$(i,j) at pixel(i,j). We filter out all pixels with VVC < 0.45. In addition, we erode the edge of our ice mask by 3 pixels in order to limit the influence of noise at the ice margin.

### Surface mass balance

We obtain surface mass balance (SMB) data from the MAR v3.10 regional climate model^7^, forced by NCEP-NCARv1 from 1992 to 2019. We limit our analyses of SMB below an ice surface elevation upper limit of 1600 m (a.s.l.). Little lake drainage occurs above this elevation^84–86^ and it has been argued that this is an approximate maximum elevation where crevasses, and thus moulins, are likely to form^87^. Whilst surface meltwater can runoff from elevations above this, surficial drainage is less likely to occur in high elevation regions due to the shallower surface slope^86^. Consequently, below this threshold elevation, we can be confident that surface meltwater drains to the ice-bed interface, and so influences ice motion. Regardless, melt at all elevations has increased from the 1958–1987 average in the period 1988–2013^87^, with the difference between the means of the period being positive at p < 0.05 from 400–2600 m (a.s.l.).

## Supplementary information

Supplementary Information.

## Data Availability

The Landsat imagery used in this study was provided by the United States Geological Survey and the European Space Agency third party missions program and are freely available.
