# Supplementary material for: Dynamic response of the Greenland ice sheet to recent cooling
Source: Sci Rep. 2020 Feb 3;10:1647. doi: 10.1038/s41598-020-58355-2 (PMC6997348; doi:10.1038/s41598-020-58355-2)
Supplement: Supplementary file 1 — Supplementary Information. [file 41598_2020_58355_MOESM1_ESM.pdf]

Supplementary Information

Dynamic response of the Greenland ice sheet to recent cooling

Joshua J. Williams[1*], Noel Gourmelen[1], Peter Nienow[1]

[1]*School of Geosciences, University of Edinburgh, Edinburgh, EH8 9XP, UK.*

*Corresponding author: j.j.williams-4@sms.ed.ac.uk

Supplementary Note 1: Detailed Methods

Remote Sensing of Ice Velocity

Repeat image feature-tracking of pairs of multispectral satellite images is often used to derive glacier surface velocity [1, 2, 3, 4]. Here, we apply these techniques to calculate ice motion from Landsat imagery. The major strength of Landsat data is the continuous archive of imagery across multiple missions, from 1972 to present, available at a synoptic scale [5, 6]. This imagery is freely available through the US Geological Survey (via https://earthexplorer.usgs.gov/) and the European Space Agency (via https://eo-sso-idp.eo.esa.int/). Given the poor availability of imagery and larger errors in georeferencing and orthorectification from Landsat 1-3, we limit our analysis to imagery from Landsat 5-8, covering the time period 1985 to present.

We calculate every Landsat path/row that intersects our study region (66.41 to 69.52 N, -51.78 to -45.45 W, Figure S1) and apply feature-tracking to all possible pairs of length 368±16 days for each path/row combination from 1985 to 2019 (Figure S2 and S3). This amounts to a total of 2665 pairs across 14 path/rows, an increase of >5 and 3 times that used by Tedstone et al [7] respectively. The temporal baseline of ~1 year is used to minimise the impact of any seasonal variability in interannual ice velocity.

Using images 16 days either side of the 368-day baseline may introduce bias whereby, for example, a 384-day period could disproportionately sample faster flow in summer [8] (or vice versa). To test the impact of a varying temporal baseline, we quantify the proportion of summer coverage along our velocity time series, displayed in Figure S4, and follow Tedstone et al [7] in using mean summer and winter GPS velocities (127.6 m yr^{-1} and 81.6 m yr^{-1} respectively) from Leverett Glacier sites [9, 10] to convert this into the potential impact upon annual velocity.

By applying a linear regression to the resultant time series, we observe no significant trend (-0.05 ± 0.03 m yr^{-2}, $p = 0.16$). We also apply a linear regression to the periods calculated by the segmented linear regression of our velocity anomaly time series, and find no significant trend introduced for any subsection of the time series (0.15 ± 0.11 m

yr^{-2}, $p = 0.21$, 0.25 ± 0.13 m yr^{-2}, $p = 0.08$, 0.25 ± 0.19 m yr^{-2}, $p = 0.22$, for segments 1 through 3 respectively). Consequently, we argue that the trends in annual ice velocity are not artefacts of a varying temporal baseline.

Joughin et al [8] used winter velocities (1st Sept - 31st May) to assess interannual change, arguing that this should minimise any seasonal variation. However, GPS data from multiple studies indicate that ice flow varies considerably through winter [9, 11, 12, 13] and so introduces problems when attempting to determine net winter velocity from a subset of winter, as the particular period sampled will impact upon the final velocity. This is especially important given the slower-flowing nature of land-terminating margins. Consequently, we argue that calculating velocities with a temporal baseline of ~1 year best captures changes in interannual ice velocity. Moreover, our temporal baseline of 352-384 days is shorter than that of Tedstone et al [7] (352-400 days), which should reduce any impact of disproportionate sampling.

The feature-tracking processing chain is explained in detail in Dehecq et al [2]. Here, we use oriented correlation, matching the feature of gradient orientation for each pixel [14], as this is found to give improved spatial coverage of the output velocity fields compared to normalised cross-correlation. Following Tedstone et al [7], we use a combination of spectral bands at wavelengths ~0.52-0.69 μm (bands 2+3 for Landsat 5 and 7, 3+4 for Landsat 8), and we enhance the images by applying a principal component analysis to these bands. Subsequently, a high-pass gaussian filter is used to enhance surface features such as crevasses and reduce the impact of temporally stable features relating to the basal topography [2, 7].

Whilst we use the same grid spacing of 8 pixels (240m) as Tedstone et al [7], we use a larger reference window size of 80 pixels (2400m). We found that this was necessary to generate velocity fields with good spatial coverage across the margin in the Leverett Glacier region, to the south of the region studied by Tedstone et al [7]. We follow Dehecq et al [2] by performing feature tracking for selected cloud-free pairs with different reference window sizes, and subsequently choosing the size at which errors over stable ground are minimised and become asymptotic as the reference window size is increased

further. The search window is automatically set based on the maximum expected displacement over the pair duration from prior velocity observations [15].

As reference images (i.e. Global Land Survey) are not available for every path/row, we apply a median coregistration to the output velocity fields in order to remove errors associated with georeferencing, whereby we calculate the median velocity over stable ground (where velocity should equal zero) and remove this from the calculated ice velocity at each pixel (following [7]). Coregistration is particularly important for slower-flowing regions as artificial offsets may contribute to a significant proportion of any change signal.

The coregistered velocity fields are then fused via a spatio-temporal median over annual or multi-year periods in order to exploit the redundancy offered by many overlapping image pairs to produce robust velocity outputs. We remove low-quality velocity estimates through filtering by a threshold signal-to-noise ratio value (snr threshold = 6). Following previous work [2, 7], we identify this threshold value by calculating the value beyond which the median absolute deviation of velocities over stable ground becomes asymptotic. For Landsat 7 and 8 data, we group velocities into 1- and 2-year periods, whereas for Landsat 5 data, we group the results into 4-year periods (as fewer pairs are available and given the difficulty in producing velocity outputs with good coverage over the southern sector of our study region). The final velocity field for each time period is composed of the median value of all velocity fields within the time period at each pixel. We use the median value as this is not sensitive to large outliers, and so can filter out the impact of any erroneous values that remained above the signal-to-noise ratio threshold. The output velocity merges are re-projected to Polar Stereographic given that the path/row combinations span several UTM zones.

To calculate the uncertainty at each pixel for the fused velocity fields, we compute a 1σ confidence interval for each component of the velocity field based on the median absolute deviation of the velocity over stable ground and the number of velocity estimates used in the calculation of the median [2]. This follows the form:

$$\sigma = \frac{k}{2} \cdot \frac{MAD}{N^{\alpha}}$$

Where MAD is the median absolute deviation over stable ground, N is the number of velocity fields used in the computation of the median velocity, σ is the 1σ confidence interval, and k and α are parameters determined for each time period from the stable ground velocity which is known to equal zero. This relationship is extrapolated on-ice using the appropriate values of MAD and N at each pixel in order to give the uncertainty of the ice velocity estimates for each time period. Following Tedstone et al [7], we discard pixels with $\sigma > 60$ m yr^{-1} from the subsequent analyses. In addition, we mask out the tidewater glaciers to the very north of the study region in order to remove any influence of ice experiencing different dynamic processes from our land-terminating results.

To compute a multi-annual ice velocity time series, we first remove all points above the 60 m yr^{-1} uncertainty threshold from each merged output. Given that the velocity fields become increasingly noisy at higher surface elevations, we use the Greenland Ice Mapping Project DEM [16] to limit our analysis to pixels below 1000 m above sea-level. As there are differences in spatial coverage between time periods, there exists a compromise to be made between spatial and temporal coverage, with the aim to assess change across many common pixels whilst retaining a high temporal resolution. We retain only the velocity fields with an area coverage above 9250 km^2. If we apply a minimum coverage threshold of 9000 km^2, we lose ~35 % of our common points (1429 km^2). Increasing the minimum coverage threshold to 9500 km^2 results in the loss of data from the time series, which increases as the threshold increases further. As such, we take 9250 km^2 as the coverage threshold which offers the best compromise between spatial and temporal coverage.

The median velocity is subsequently calculated across the 71703 pixels common to all the remaining merged velocity fields (Figure S5) in order to avoid spatial bias influencing the change signal. By multiplying this by the area of an individual pixel (240*240 m), we calculate a common areal coverage of 4130 km^2. This represents an order of magnitude

increase in the number of common pixels, and thus common area, between velocity merges when compared to Tedstone et al [7]. The associated uncertainty is estimated across the same pixels in the form:

$$\sqrt{\sum_{i=1}^{N} \sigma_i^2 / N}$$

Anomaly-based Time Series

Time series of ice velocity are commonly displayed in terms of difference in velocity magnitude [1, 7, 17, 18]. Recent work [19] indicates that velocity magnitude has a biased mean, with this bias increasing with the standard deviation of the velocity components (and so with noise). Consequently, this causes an artificial negative velocity trend, particularly so in slow-flowing sectors and where velocities are not significantly larger than noise, when calculating a multi-mission time series.

To mitigate this effect, we follow the velocity anomaly approach of Dehecq et al [19]. First, we calculate a mean of all velocity pairs covering the period 1992-2017 described as V_0. The velocity anomaly is defined as the value of the difference vector $V_t - V_0$ projected on the mean velocity vector:

$$dv = \frac{(V_t - V_0) \cdot V_0}{\|V_0\|} = \frac{(V_{x,t} - V_{x,0})\, V_{x,0} + (V_{y,t} - V_{y,0}) V_{y,0}}{\|V_0\|}$$

The result of this approach is to centre the noise distribution symmetrically around zero (Figure S6) such that there is no bias in the mean value, removing any artificial slowdown trends due to variability in noise magnitude between sensors [19]. The resultant velocity anomaly fields are displayed in Figure S7.

Calculating a Long-Term Ice Velocity Trend

To assess the long-term trends in our time series, we first compute a simple linear regression through the data, which gives a velocity change of -0.72 ± 0.08 m yr^{-2} from 1992 to 2019 ($R^2 = 0.70$, $p < 0.01$). Whilst a long-term slowdown is clear from the time series, it also shows a period of sustained high velocity, followed by a period of slowdown, and then an apparent stabilisation. Consequently, we test whether the data can be divided into three statistically different segments (Figures S8, S9 and S10).

We use the non-parametric Mann-Whitney Wilcoxon Test to determine whether the medians of independent populations are similar. We split the times series into two segments, 1992-2012 and 2002-2019, and test all possible pairs of breakpoints at 0.2-year intervals along each. We also compute the root mean squared error (RMSE) for each pair. This describes how close the observed data points are to the modelled fit, with lower RMSE indicating a better fit to the data. For the first period, we find that for breakpoints between 1996 and 2011, the null hypothesis of equal medians can be rejected (Figure S8C). This means that pre- and post-1996, populations are significantly different with 95% confidence. From Figure S8B, we observe that for pre- and post-2000 populations, the null hypothesis can be rejected with 99 % certainty ($p = 0.01$). The minimum RMSE (Figure S8A) is during 2003, however there is a region of low RMSE from 2002-2005 which arises from a gap in our dataset. From this, we take a breakpoint of 2003 given that this year has the lowest RMSE (and so the best fit to the velocity anomaly time series) and separates two populations with statistically different medians at the 99 % confidence interval.

For the second period (Figure S9), the null hypothesis of equal medians can be rejected for breakpoints between 2006 and 2012. A minimum in RMSE is observed in 2012, and so we take this as the second breakpoint in our time series, which also separates two populations with statistically different medians ($p=0.01$). As a result, we select the pair of breakpoints with the lowest RMSE, that are statistically significant with 99 % certainty, which gives breaks at 2003 and 2012, and an improved R^2 of 0.85.

The RMSE of each pair of breakpoints tested with the Mann-Whitney Wilcoxon test are displayed in Figure S10. Whilst the breakpoints with the lowest RMSE are 2003 and 2012, there is a large area of low RMSE for pairs of breakpoints between ~2002-2005 and ~2010-2014, although the null hypothesis cannot be rejected for a breakpoint between 2013-2014. Consequently, whilst we cannot conclusively state any exact dates for the pair of breakpoints in the time series, we conclude that our ice velocity time series can be divided into three statistically different segments, with the breakpoints during the periods 2002-2005 and 2010-2012.

Impacts of Calculating a Multi-Mission Trend

It is important to consider that the second break in our time series (Figure 3) corresponds closely with the change in sensor between Landsat 7 and Landsat 8. Whilst numerous studies have calculated velocity trends using multiple sensors, recent research [8, 19] indicates that biases exist between missions and sensors. This is especially important to consider where breakpoints in a time series coincide with changes in sensor or mission, as it cannot initially be ruled out that a change in trend is simply the result of biases between sensors/missions. Over time, the Landsat missions have improved in radiometric quality, decreasing both noise and the number of potential mismatches [19]. In addition, Landsat 8 has a 12-bit radiometric quantisation, as opposed to 8-bit for Landsat 5 and 7. Whilst this enables the tracking of subtle surface features and the extension of offset-tracking to regions beyond crevassed areas [3], ice features that were not previously visible can now be tracked, leading to an apparent speed-up [19]. Sensor resolution has also improved from 30*30 m to 15*15 m with the introduction of the panchromatic band (band 8) in Landsat 7 and 8. This can lead to biases in high strain regions [8], where higher resolution can lead to increased tracking of sharp features, and on narrow glaciers where coarser resolution imagery is more likely to lock onto stable ground.

In the Himalayas, it has been possible to correct for this as sufficient Landsat 5 imagery exists alongside Landsat 7 imagery, and Landsat 7 imagery is available post-2013 such that pairs of Landsat 5 and Landsat 7, as well as Landsat 7 and Landsat 8, scenes can be compared over essentially the same time period [19] in order to calculate the offset

between missions. This work indicates that velocities obtained from Landsat 8 scenes are higher than those from Landsat 7 scenes, which are in turn greater than those from Landsat 5 scenes [19]. These biases are largely < 5 m yr^{-1}, however can reach as high as 10–15 m yr^{-1} for some glaciers [19, Figures S12 and S13]. Whilst insufficient imagery is available to calculate a similar correction for Greenland (or Antarctica), were we to apply a correction to account for biases between sensors, this would be added to velocities calculated from Landsat 5 and 7 and so would not change our conclusions. We would instead observe a slight decreasing trend from the early 1990s through the early 2000s, followed by a steeper slowdown from ~2003 to 2012, after which velocities would return to a more stable trend.

As biases between missions are mostly located on narrow glaciers and at high elevations, we would expect these biases to be considerably reduced in our study region. In order to remove any biases resulting from changes in sensor resolution, we limit our analysis to the same 30*30 m spatial resolution and spectral band combination (~0.52-0.69 µm) for all three sensors. Moreover, the ice velocity anomaly approach acts to limit the influence of reductions in sensor noise over time. Consequently, we are confident that our dataset is robust against any biases introduced by using multiple sensors.

Statistical Links between Ice Velocity and Surface Melt Production

Previous work argues for a statistical link between antecedent melt production and ice motion, whereby including the four prior years of melt production is suggested to explain 44-50 % of the observed ice motion [7]. However, subsequent work has argued that the fact that an improved correlation is observed when multiple years of runoff are included is “an expected outcome of analysing two variables with long-term temporal trends, even if the mechanism generating these trends is unrelated to the annual variability” [20, p. 11301]. By repeating the analysis presented in [7], we show that R^2 tends towards 1 as more antecedent melt years are included (Figure S12). As such, it is not trivial to statistically link trends in ice motion and surface melt production.

To investigate the link between year-to-year variations in melt production and ice velocity, we detrend both time series and apply a linear regression to the resultant detrended time series. The segmented linear regression presented in Figure 3 was used to detrend the ice velocity anomaly time series. Following this, we detrended the melt production anomaly by calculating separate linear regressions for the same three time periods.

Our detrended regression gives an R^2 of 0.08 (p = 0.11), suggesting the year-to-year variation in melt production has no significant impact on year-to-year variations in ice velocity (Figure S13).

Spatial Trends in Ice Velocity

To investigate spatial trends in velocity change across our study region, we construct a change map displaying the percentage ice velocity change between the following periods; 1992-2003 and 2003-2012, 2010-2012 and 2017-2019. Median ice velocity for each period was calculated through fusing all of the constituent velocity fields via a spatio-temporal median, as described previously. We calculate uncertainty of the percentage change through a linear combination of the uncertainties of each period in the form:

$$\sqrt{{c_1}^2 + {c_2}^2}$$

Where c_1 is the first period (i.e. 1992-2003) and c_2 is the second period (i.e. 2003-2012).

As with our time series analysis, we remove pixels with uncertainty greater than 60 myr^{-1}. In addition, we also filter by Velocity Vector Coherence (VVC) [2], which describes the alignment of velocity vectors. This follows the form:

$$VVC(i,j) = \frac{\left\| \sum_{t \in T} \underset{V}{\rightarrow} (i,j,t) \right\|}{\sum_{t \in T} \left\| \underset{V}{\rightarrow} (i,j,t) \right\|}$$

Where T is the set of N velocity estimates V(i,j,t) merged to obtain the median velocity $\bar{V}$(i,j) at pixel(i,j). We filter out all pixels with VVC < 0.45. In addition, we erode the edge of our ice mask by 3 pixels in order to limit the influence of noise at the ice margin.

We then calculated the median percentage change in ice velocity across the remaining pixels for the full study region, and across bands of ice thickness (200 m bands) from BedMachine v3 [21, 22].

Surface Mass Balance

We obtain surface mass balance (SMB) data from the MAR v3.10 regional climate model [23], forced by NCEP-NCARv1 from 1992 to 2019. We limit our analyses of SMB below an ice surface elevation upper limit of 1600 m (a.s.l.). Little lake drainage occurs above this elevation [24, 25, 26], and it has been argued that this is an approximate maximum elevation where crevasses, and thus moulins, are likely to form [27]. Whilst surface meltwater can runoff from elevations above this, surficial drainage is less likely to occur in high elevation regions due to the shallower surface slope [26]. Consequently, below this threshold elevation we can be confident that surface meltwater drains to the ice-bed interface, and so can influence ice motion. Regardless, melt at all elevations has increased from the 1958-1987 average in the period 1988-2013 [27], with the difference between the means of the period being positive at $p<0.05$ from 400-2600 m (a.s.l.).

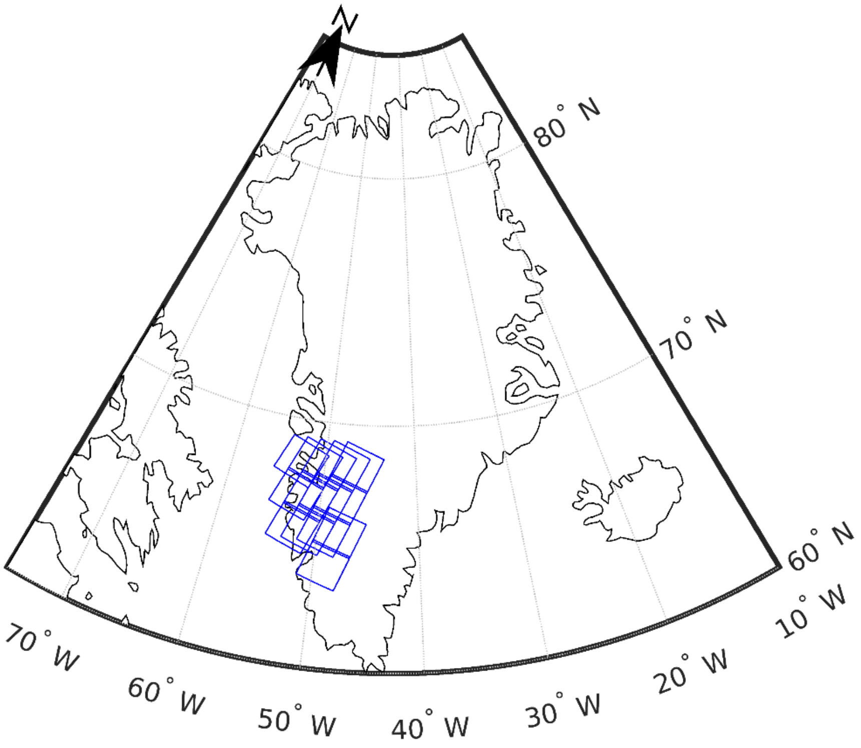

Supplementary Figure 1. *Map displaying the coverage of the WRS2 path/row combinations used in this study. Each path/row is displayed as a blue square.*

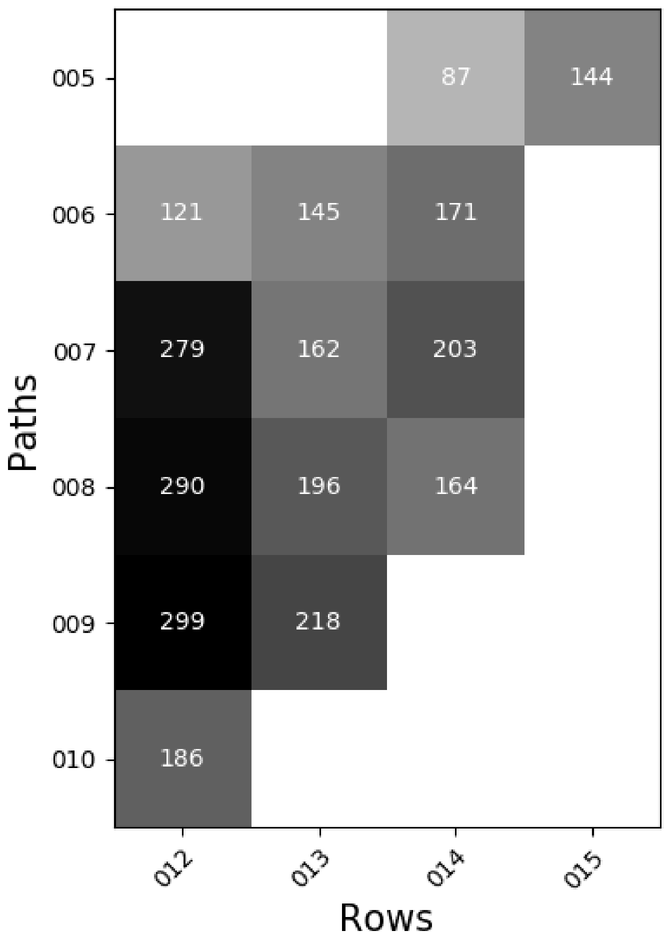

Supplementary Figure 2. *Heatmap displaying the number of image pairs for each WRS2 path/row combination. Darker shading denotes more image pairs, with the exact number noted in white for each path/row.*

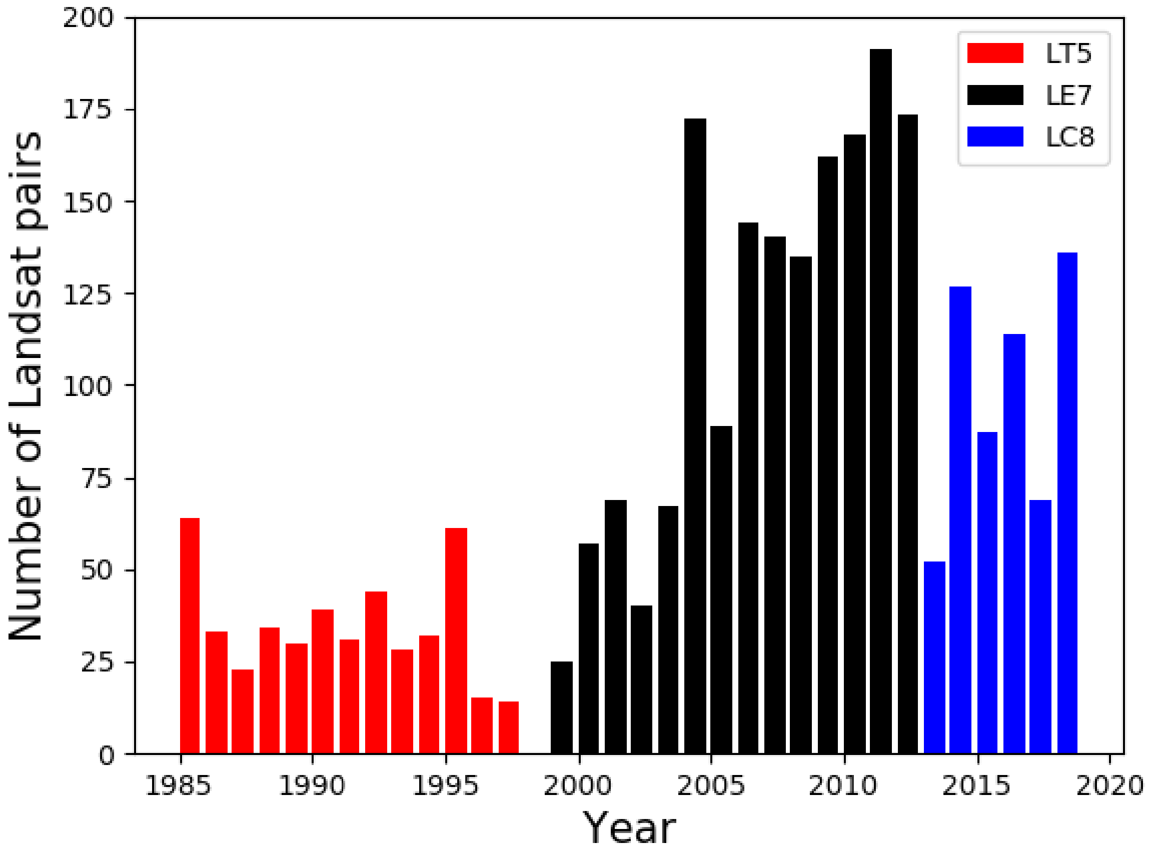

Supplementary Figure 3. *Bar chart displaying the number of Landsat image pairs for each year. Image pairs from Landsat 5, 7 and 8 scenes are displayed in red, black and blue bars respectively.*

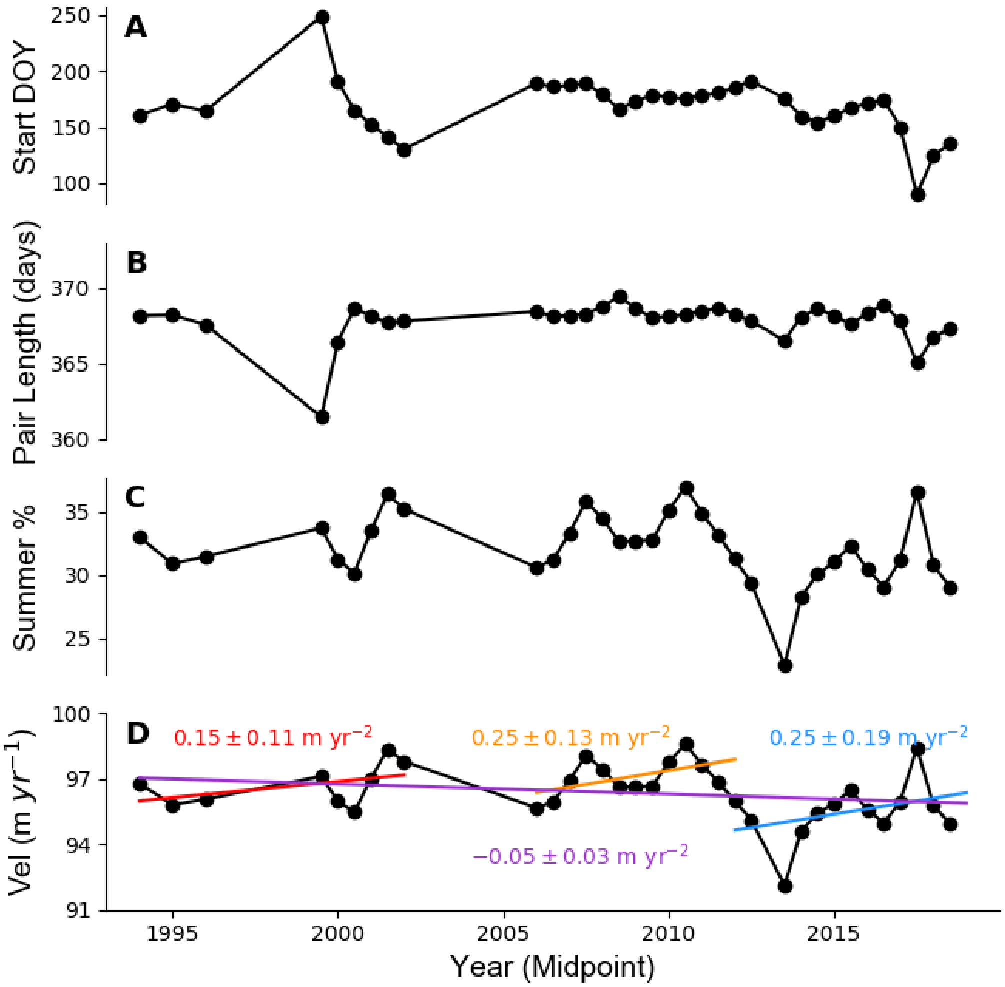

Supplementary Figure 4. *Temporal baseline analysis of the Landsat image pairs used in each period of the time series in Figure 1 (with the year displayed as the midpoint of the time period).* ***(A)*** *The average start day-of-year (DOY) of all image pairs in each period,* ***(B)*** *the average baseline duration of all pairs,* ***(C)*** *the percentage of the baseline duration that falls within summer, defined as May 1*st *– August 31*st*,* ***(D)*** *the annual velocity expected in the ablation zone of the Leverett Glacier catchment (see Methods), based upon the percentage of summer and winter coverage, and the average baseline duration for each period. Linear regressions for the entire time series (purple), as well as for each segment (red, orange, blue), are displayed.*

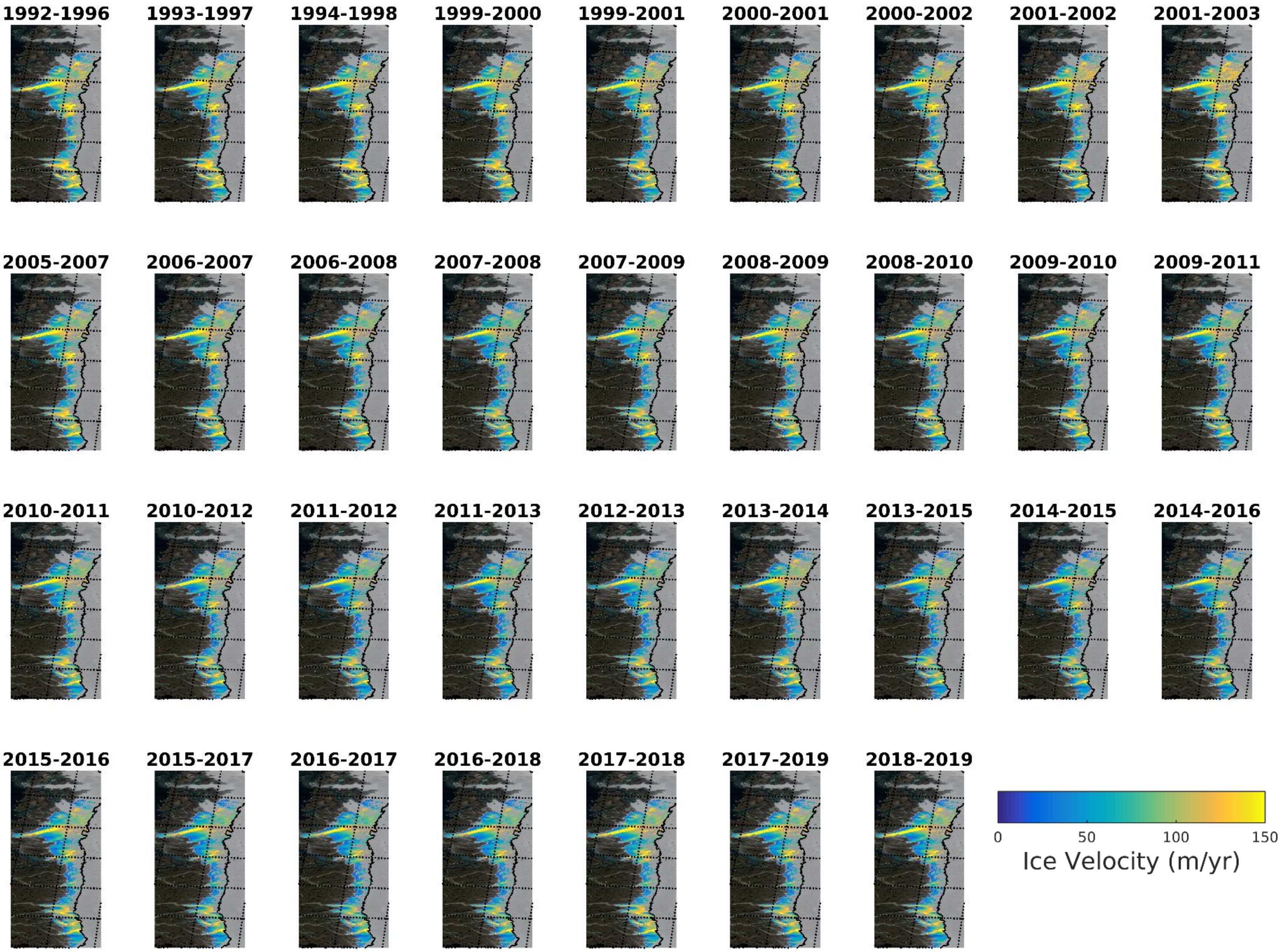

Supplementary Figure 5. *Ice velocities (m yr^{-1}) during each time period. Only the pixels common to all periods are displayed. The black contour displays 1000 m surface elevation, and the velocity fields are displayed over a MODIS (Terra) corrected reflectance image from EOSDIS NASA Worldview (https://worldview.earthdata.nasa.gov/).*

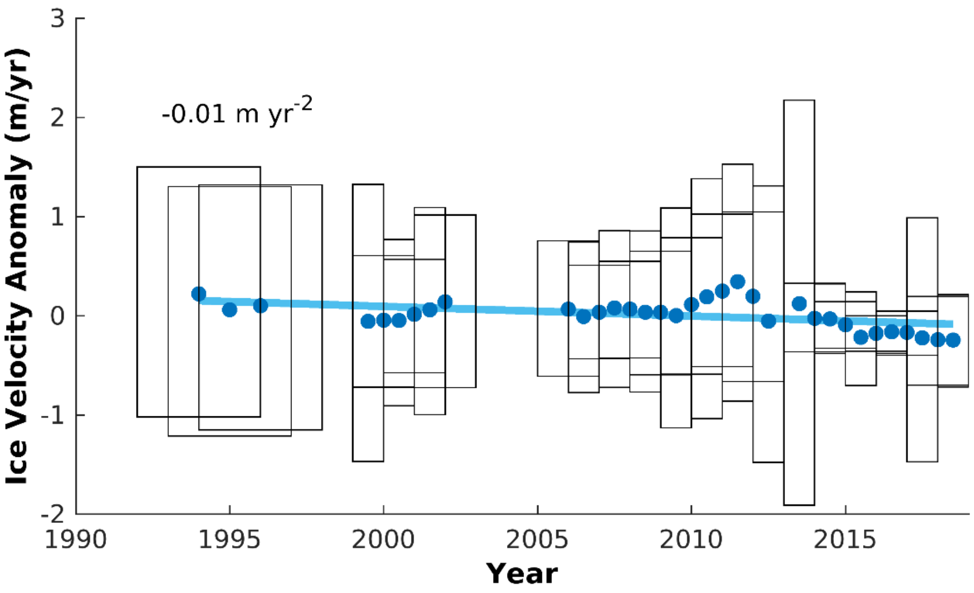

Supplementary Figure 6. *Off-ice velocity anomaly (m yr^{-1}) time series. The width of each box refers to the time period covered by that data point, and the height of the box displays the interquartile range. The light blue line shows a linear regression fit to the data.*

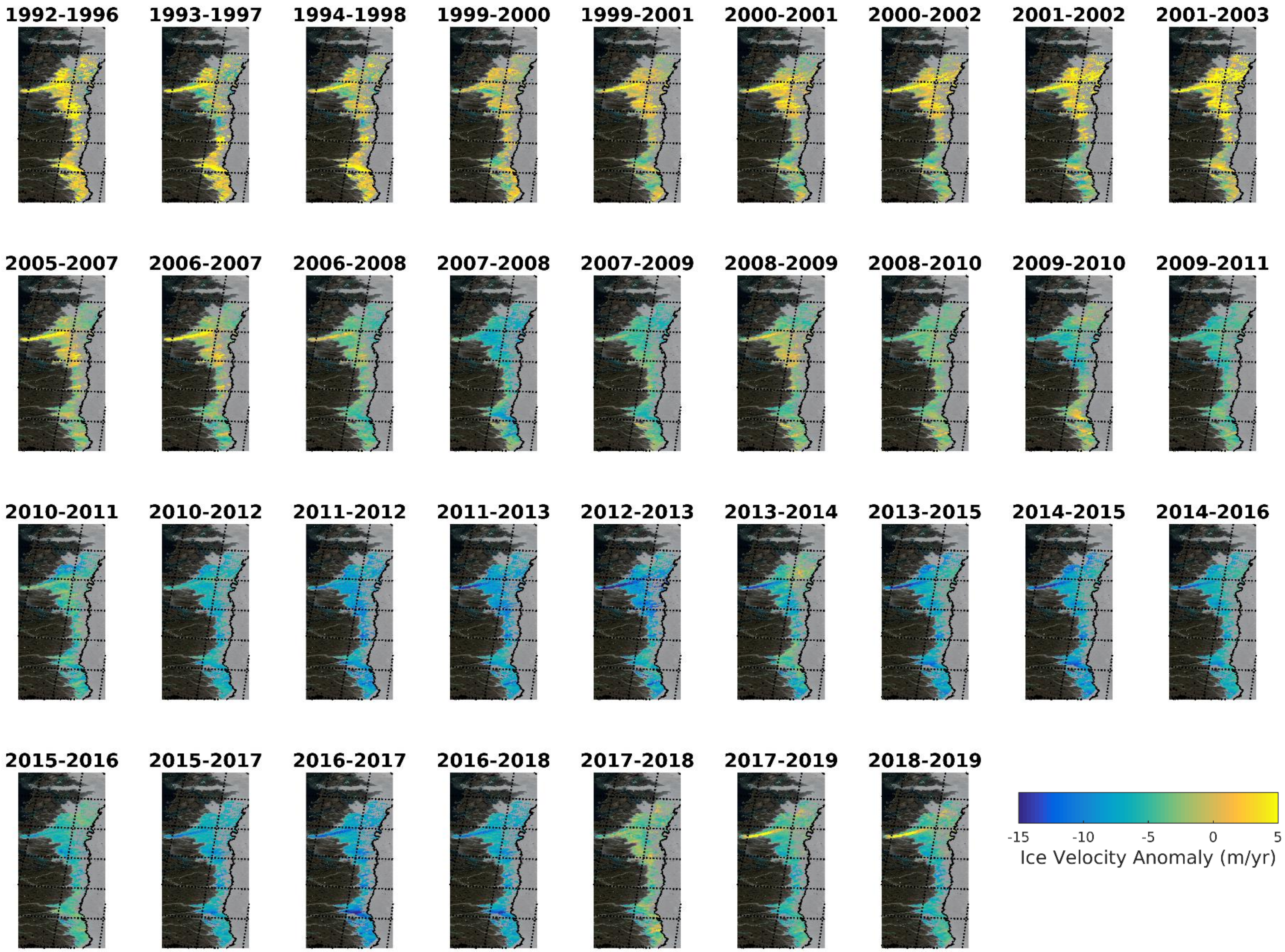

Supplementary Figure 7. *Ice velocity anomalies (m yr^{-1}) during each time period. Only the pixels common to all periods are displayed. The black contour displays 1000 m surface elevation, and the velocity fields are displayed over a MODIS (Terra) corrected reflectance image from EOSDIS NASA Worldview (https://worldview.earthdata.nasa.gov/).*

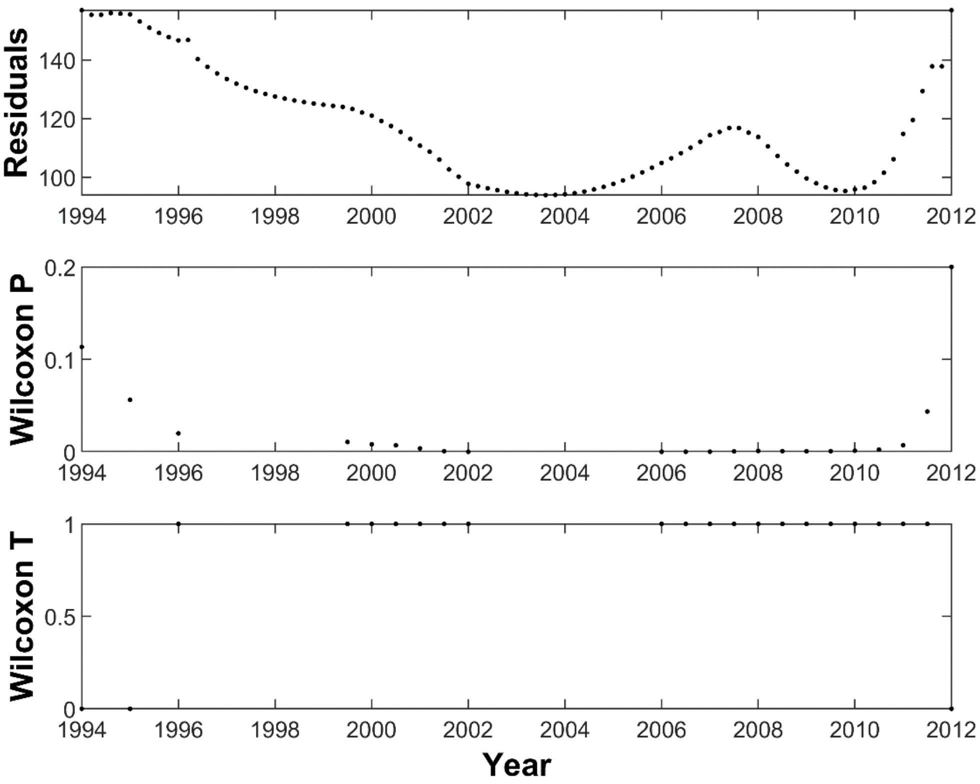

Supplementary Figure 8. *Statistical significance of two periods of ice motion between 1994 and 2012.* ***(A)*** *Residuals displayed as the RMSE of a two-trend model fitted to the ice velocity anomaly data at each tested breakpoint.* ***(B)*** *Probability that the observed result occurred by chance if the null hypothesis that medians are equal is true.* ***(C)*** *Hypothesis test of the Wilcoxon rank sum test for equal medians, testing that the probability of two populations are similar at the 95 % confidence interval. 0 denotes that the hypothesis of equal medians cannot be rejected, 1 denotes that the hypothesis of equal medians can be rejected.*

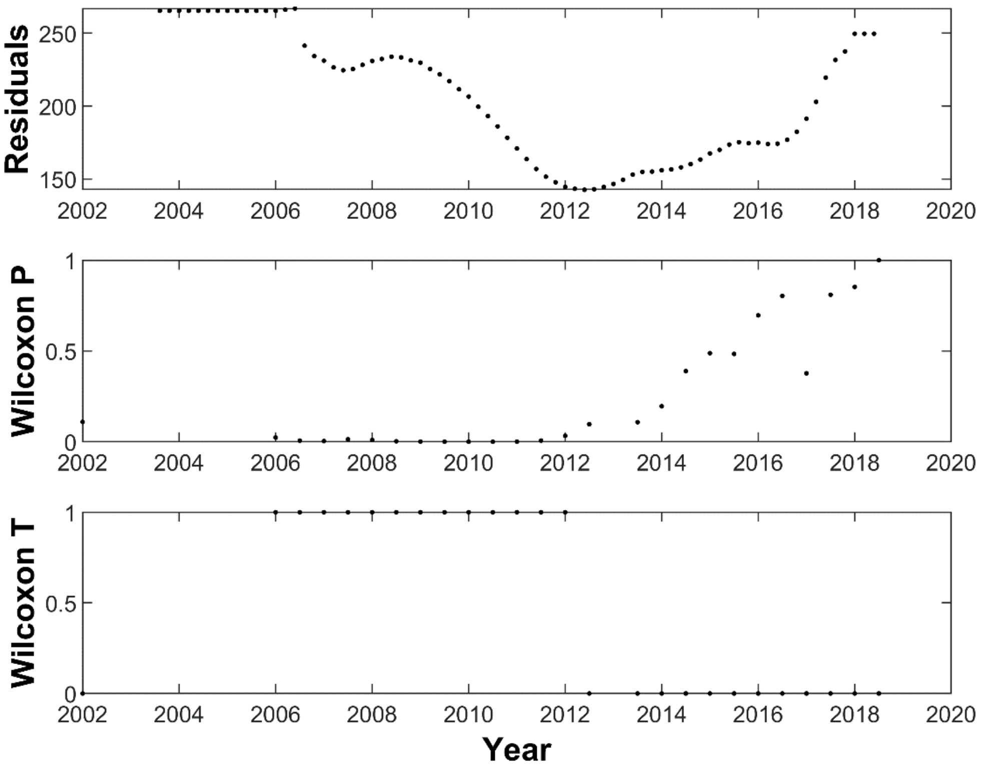

Supplementary Figure 9. *Statistical significance of two periods of ice motion between 2002 and 2019.* ***(A)*** *Residuals displayed as the RMSE of a two-trend model fitted to the ice velocity anomaly data at each tested breakpoint.* ***(B)*** *Probability that the observed result occurred by chance if the null hypothesis that medians are equal is true.* ***(C)*** *Hypothesis test of the Wilcoxon rank sum test for equal medians, testing that the probability of two populations are similar at the 95 % confidence interval. 0 denotes that the hypothesis of equal medians cannot be rejected, 1 denotes that the hypothesis of equal medians can be rejected.*

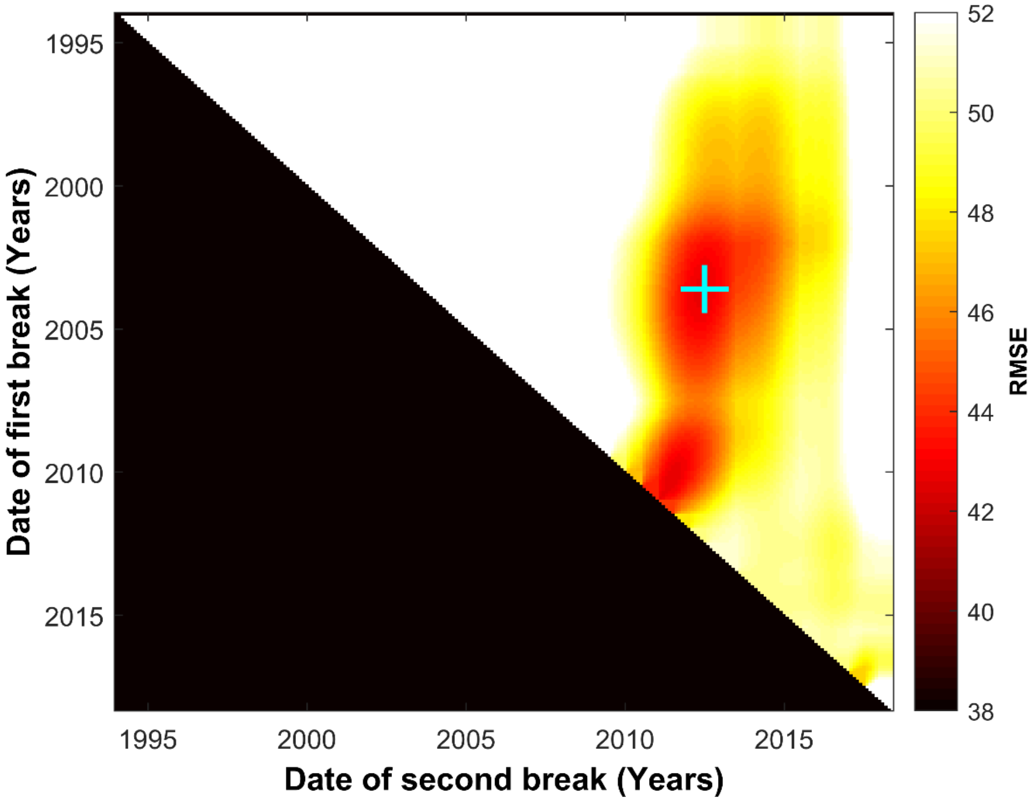

Supplementary Figure 10. *Hypothesis test of the Wilcoxon rank-sum test for statistically different medians between three periods of ice velocity anomalies. The coloured half shows the root mean square errors (RMSE) of a three-segment linear model fitted to all possible combinations of two break dates at intervals of 0.2 years. The centre of the cyan cross denotes the location of the pair of breakpoints with the lowest combined RMSE. The black area is shaded for simplicity as it mirrors the coloured sector.*

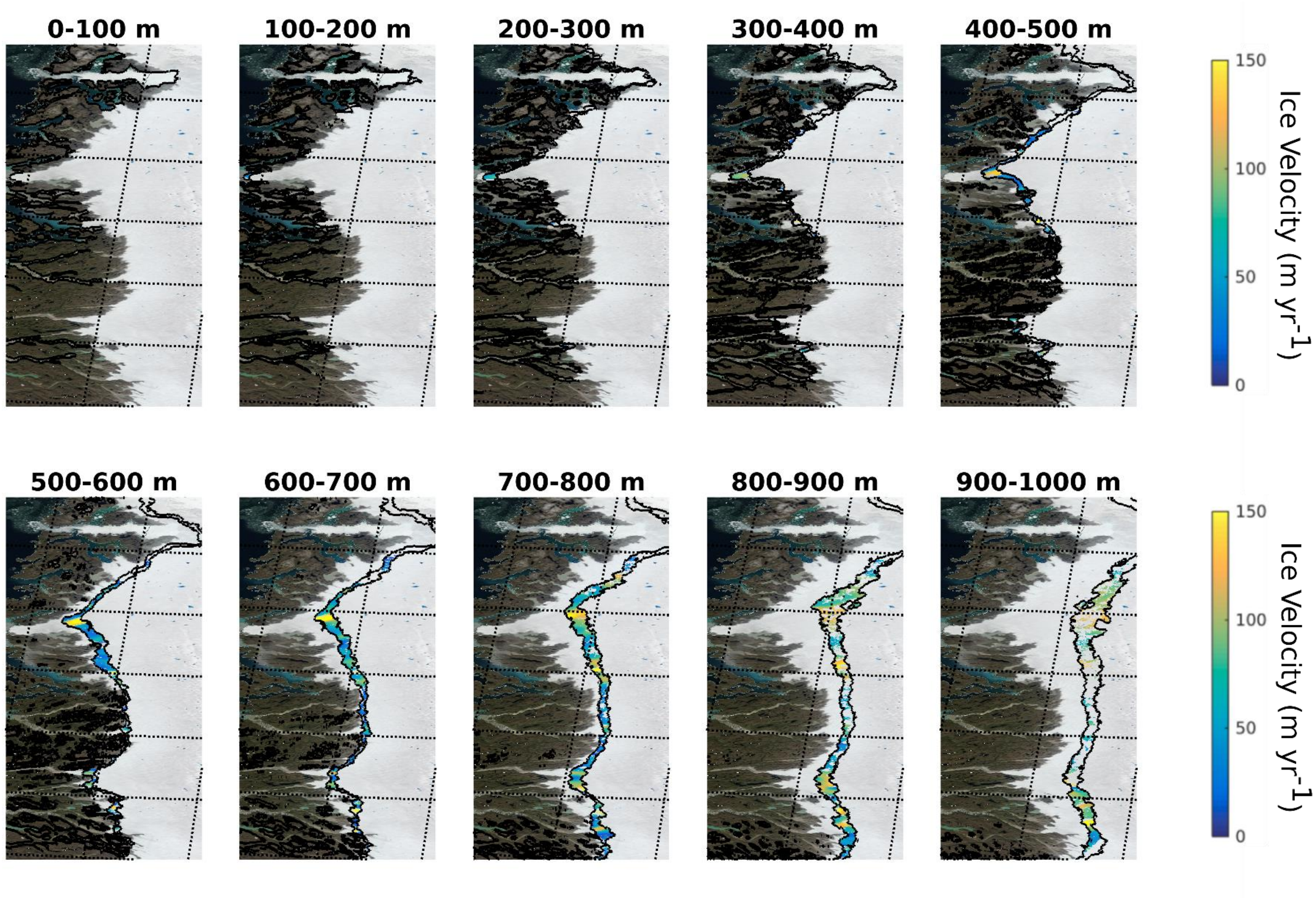

Supplementary Figure 11. *Ice velocity (m yr^{-1}) coverage across each 100 m surface elevation band. Only the pixels common to all periods are displayed. The displayed ice velocities are from the 2013-2015 merged velocity field. The base map is a MODIS (Terra) corrected reflectance image from EOSDIS NASA Worldview (https://worldview.earthdata.nasa.gov/).*

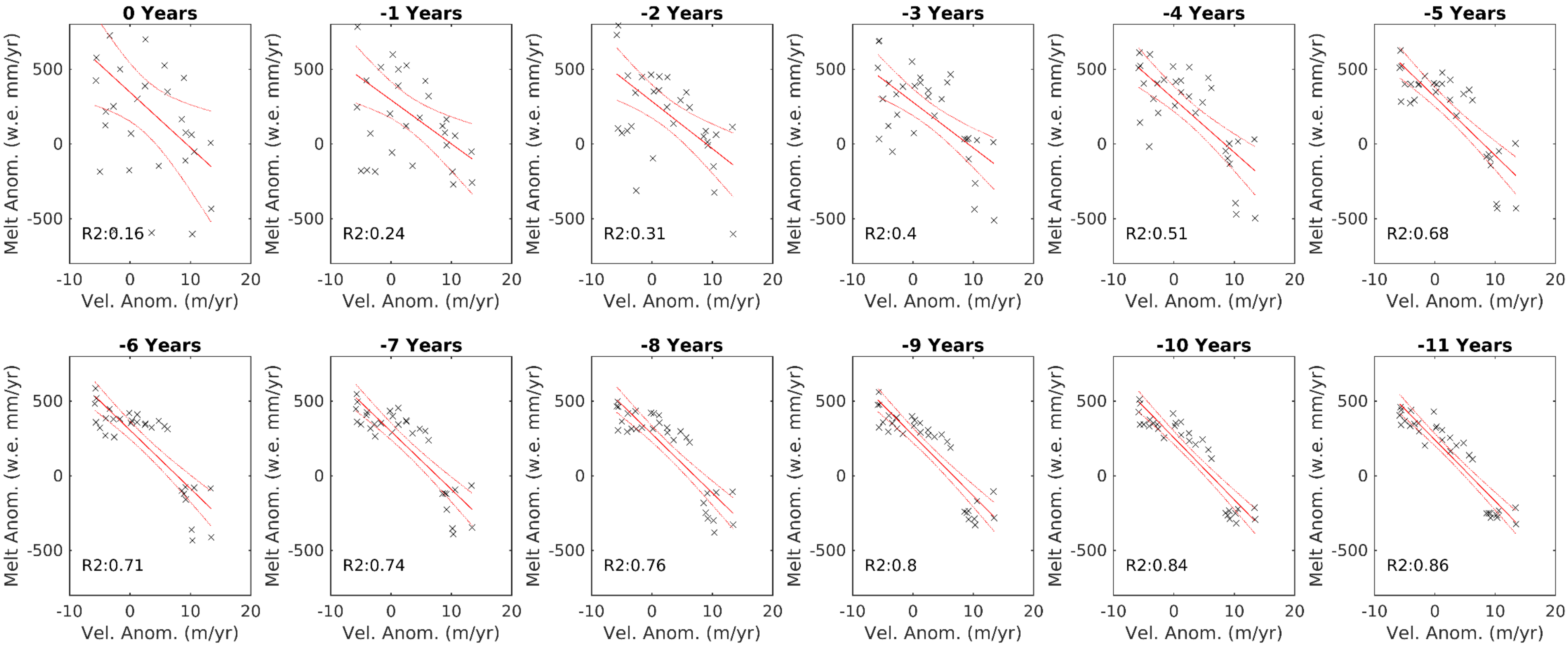

Supplementary Figure 12. *Linear regressions between ice velocity anomalies (m yr^{-1}) and antecedent surface melt production anomalies (w.e. mm yr^{-1}). The title of each plot refers to the number of years of melt data prior to each velocity point that were included. The R^2 value of each regression is displayed within each subplot.*

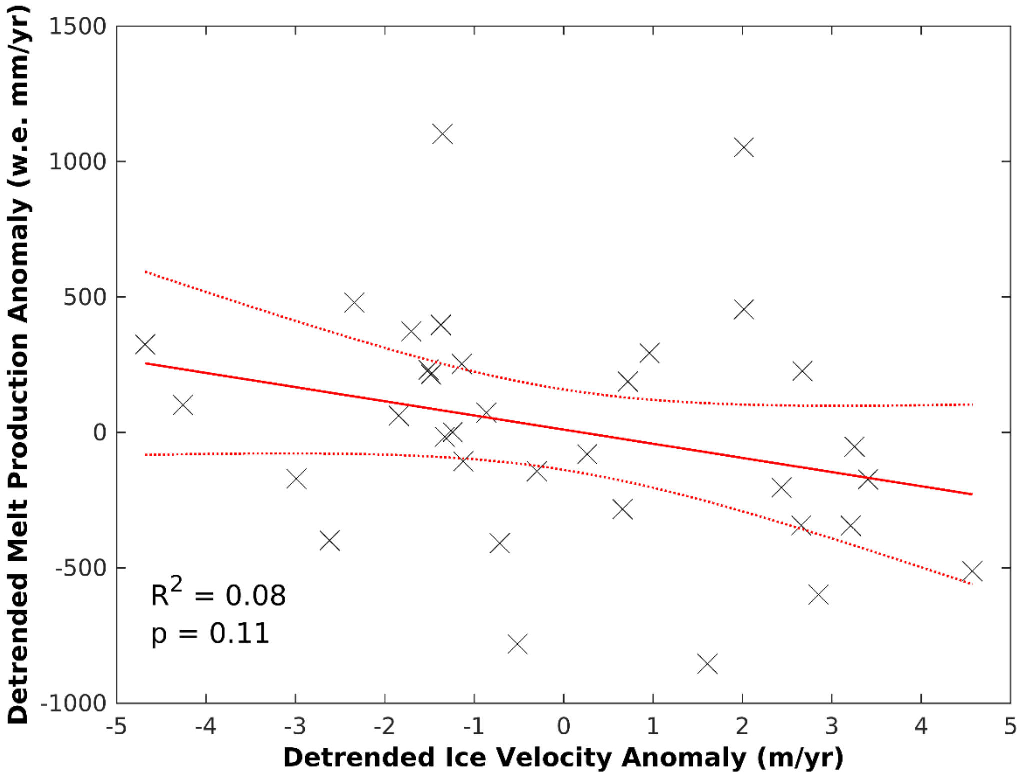

Supplementary Figure 13. *Linear regression between detrended ice velocity anomalies (m/yr) and detrended melt production anomalies (mm/yr). The regression is displayed by the solid red line, with confidence bounds displayed by the dotted red lines.*

Supplementary References

1. Heid, T. & Kääb, A. Evaluation of existing image matching methods for deriving glacier surface displacements globally from optical satellite imagery. *Remote Sensing of Environment* 118:339–355 (2012).

2. Dehecq, A., Gourmelen, N. & Trouve, E. Deriving large-scale glacier velocities from a complete satellite archive: Application to the Pamir–Karakoram–Himalaya. *Remote Sensing of Environment* 162:55–66 (2015).

3. Fahnestock, M. et al. Rapid large-area mapping of ice flow using Landsat 8. *Remote Sensing of Environment* 185:84–94 (2016).

4. Jeong, S., Howat, I.M. & Ahn, Y. Improved Multiple Matching Method for Observing Glacier Motion With Repeat Image Feature Tracking. *IEEE Transactions on Geoscience and Remote Sensing* 55(4):2431–2441 (2017).

5. Pope, A. et al. Open Access Data in Polar and Cryospheric Remote Sensing. *Remote Sensing* 6(7):6183–6220 (2014).

6. Wulder, M.A. et al. The global Landsat archive: Status, consolidation, and direction. *Remote Sensing of Environment* 185:271–283 (2016).

7. Tedstone, A.J. et al. Decadal slowdown of a land-terminating sector of the Greenland Ice Sheet despite warming. *Nature* 526(7575):692–695 (2015).

8. Joughin, I., Smith, B.E. & Howat, I. Greenland Ice Mapping Project: ice flow velocity variation at sub-monthly to decadal timescales. *The Cryosphere* 12(7):2211–2227 (2018).

9. Sole, A. et al. Winter motion mediates dynamic response of the Greenland Ice Sheet to warmer summers. *Geophysical Research Letters* 40(15):3940–3944 (2013).

10. Tedstone, A.J. et al. Greenland ice sheet motion insensitive to exceptional meltwater forcing. *Proceedings of the National Academy of Sciences* 110(49):19719–19724 (2013).

11. Joughin, I. et al. Seasonal speedup along the Western flank of the Greenland Ice Sheet. *Science* 320(5877):781-783 (2008).

12. Colgan, W. et al. The annual glaciohydrology cycle in the ablation zone of the Greenland ice sheet: Part 2. Observed and modeled ice flow. *Journal of Glaciology* 58(207):51–64 (2012).

13. van de Wal, R.S.W. et al. Self-regulation of ice flow varies across the ablation area in south-west Greenland. *The Cryosphere* 9(2):603–611 (2015).

14. Fitch, A.J., Fitch, A.J., Kadyrov, A., Christmas, W.J. & Kittler, J. Orientation correlation. *Br Mach Vis Conf* 1:133-142 (2002).

15. Joughin, I., Smith, B.E., Howat, I.M., Scambos, T. & Moon, T. Greenland flow variability from ice-sheet-wide velocity mapping. *Journal of Glaciology* 56(197):415–430 (2010).

16. Howat, I.M., Negrete, A. & Smith, B.E. The Greenland Ice Mapping Project (GIMP) land classification and surface elevation data sets. *The Cryosphere* 8(4):1509–1518 (2014).

17. Moon, T., Joughin, I. & Smith, B. Seasonal to multiyear variability of glacier surface velocity, terminus position, and sea ice/ice mélange in northwest Greenland. *Journal of Geophysical Research: Earth Surface* 120(5):818–833 (2015).

18. Lemos, A. et al. Ice velocity of Jakobshavn Isbræ, Petermann Glacier, Nioghalvfjerdsfjorden, and Zachariæ Isstrøm, 2015–2017, from Sentinel 1-a/b SAR imagery. *The Cryosphere* 12(6):2087–2097 (2018).

19. Dehecq, A. et al. Twenty-first century glacier slowdown driven by mass loss in High Mountain Asia. *Nature Geoscience* 12(1):22–27 (2019).

20. Stevens, L.A. et al. Greenland Ice Sheet flow response to runoff variability. *Geophysical Research Letters* 43(21):11,295-11,303 (2016).

21. Morlighem, M. et al. BedMachine v3: Complete Bed Topography and Ocean Bathymetry Mapping of Greenland From Multibeam Echo Sounding Combined With Mass Conservation. *Geophysical Research Letters* 44(21):11,051-11,061 (2017).

22. Morlighem, M. et al. 2017 to present, updated 2018. *IceBridge BedMachine Greenland, Version 3*. [Bed Elevation, Ice Thickness]. Boulder, Colorado USA. NASA National Snow and Ice Data Center Distributed Active Archive Center. doi: https://doi.org/10.5067/2CIX82HUV88Y. [01/02/2019].

23. Fettweis, X. et al. Reconstructions of the 1900–2015 Greenland ice sheet surface mass balance using the regional climate MAR model. *The Cryosphere* 11(2):1015–1033 (2017).

24. Howat, I.M., de la Peña, S., van Angelen, J.H., Lenaerts, J.T.M. & van den Broeke, M.R. Brief Communication: Expansion of meltwater lakes on the Greenland Ice Sheet, *The Cryosphere* 7(1):201–204 (2013).

25. Johansson, A.M., Jansson, P. & Brown, I.A. Spatial and temporal variations in lakes on the Greenland Ice Sheet. *Journal of Hydrology* 476:314–320 (2013).

26. Selmes, N., Murray, T. & James, T.D. Characterizing supraglacial lake drainage and freezing on the Greenland Ice Sheet. *The Cryosphere Discusions* 7(1):475–505 (2013).

27. Poinar, K. et al. Limits to future expansion of surface-melt-enhanced ice flow into the interior of western Greenland. *Geophysical Research Letters* 42(6):1800–1807 (2015).
